# The Next Step at the "London"

**Published:** 1921-05-21

**Authors:** 


### The Hospital

The Workers' Newspaper of Administrative Medicine and Institutional Life. Administration. National Insurance and Health.

No. 1824, Vol. LXX. SATURDAY, MAY 21, 1921. PRICE TWOPENCE

### THE NEXT STEP AT THE "LONDON."

The news of last week that the London Hospital has decided to close a large number of its beds must seem to Lord Knutsford but the realisation of the forebodings which he expressed a year ago. He will feel that the public was warned, and that he and his committee have exhausted all expedients, and that, notwithstanding these warnings and efforts, the public conscience has remained unquickened to the needs of the situation. It remains to be seen, however, what the effect of the decision will be. We confess to disappointment that at present it has evoked no response, and that we go to press again without news of anybody coming to the rescue. If private benefactors are still conspicuous by their absence, there are two bodies which can hardly be unmoved. The first of these is Lord Cave's Committee, whose recommendations could hardly receive stronger emphasis than the closing of about a quarter of the beds in the best-known hospital in London. The recommendations of such committees are often disregarded, but a stern fact like this cannot be passively endured. The second body which must be affected is the Approved Societies, who have such an opportunity as can hardly recur of silencing all criticisms and ending all doubts among themselves by a common decision to allot a generous part of their surplus to the hospitals. A precedent has been set and a gratifying example given already by one of their number. Is it too much to suggest that the patients on the long waiting list of the London Hospital, now so sadly increased, be invited to approach their respective societies for assistance on the ground that they cannot gain the treatment they need unless such help is given? If such action were generally taken, it would be tantamount to a vote in favour of this policy, beside being to the interest of the insured; since of what use would the additional benefits (suggested for the surplus) be if the primary benefit of institutional treatment is now unobtainable?

Notwithstanding the coal strike and its certain effects on prices and unemployment, the day of war prices seems to be on the decline, and, therefore, to save the London Hospital now may be to save it at the height of its difficulties. It is true that in the future a modification of the former methods of raising revenue may be expected to prevail (see p. 124), but, even so, the voluntary system in London is admittedly strong enough to bear a large part of the post-war burden; and what the London Hospital needs now is a definite degree of supplemental help. "We have done our best," Lord Knutsford can say; "what are those who appointed Lord Cave's Committee going to do about it?"

